# Disability among elderly rural villagers: report of a survey from Gonoshasthaya Kendra, Bangladesh

**DOI:** 10.1186/1471-2458-12-379

**Published:** 2012-05-25

**Authors:** Nicola Cherry, Morshed Chowdhury, Rezaul Haque, Corbett McDonald, Zafrullah Chowdhury

**Affiliations:** 1 University of Alberta, 5-30 University Terrace, 8303-112 St, Edmonton, AB, Canada, T6G 1K4; 2 Community-based Medical College, Gonoshasthaya Kendra, Saver, Bangladesh; 3 Gonoshasthaya Kendra, Saver, Bangladesh; 4 Imperial College, London, UK; 5 Gonoshasthaya Kendra, Dhaka, Bangladesh

## Abstract

**Background:**

The study was set up to identify the extent and nature of difficulty with activities of daily living (disabilities) among elderly village residents of Bangladesh, to describe help currently given and to identify possible interventions. It was carried out at Gonoshasthaya Kendra (GK), a community development organization responsible for the health care of 600 villages with a population of some 1.5 million.

**Methods:**

A survey card was designed and piloted using 12 questions on disability, elaborated from the Washington Group Disability questions, together with a checklist of health problems. A survey was carried out in 2010 in 535 villages under the care of GK since 2005, with village paramedics interviewing residents believed to be age 60 years or older. Respondents were matched where possible to data from the 2005 GK household census, giving data on education, occupation, socioeconomic group and smoking habit.

**Results:**

Survey cards were completed for 43417 residents of which 17346 were matched to residents recorded in the GK census as born ≤ 1945. The proportion reporting ‘much difficulty’ on one or more functional capacities increased steadily with age, reaching 55% (1796/3620) among those ≥ 85 years. Difficulties most frequently reported were lifting and carrying, vision and going outside the home. At all ages women were more likely to report ‘much difficulty’ than men (OR = 1.43 (1.35 to 1.48)), with widows and the illiterate at greater risk. Health problems, particularly hemiplegia, resting tremor, urinary incontinence and depression were strongly related to the 12 disabilities assessed. Help came almost entirely from family members; of 11211 villagers with ‘much difficult’ on at least one functional capacity, only 15 reported getting help outside the family.

**Conclusions:**

Disabled elderly residents were dependent on the family for help but, with family cohesiveness under threat from migration to the city, there is a pressing need for the development and critical evaluation of community-based interventions designed specifically for the elderly in poor rural societies. New approaches to training and practice will be needed to integrate such disability management into primary care.

## Background

Bangladesh is a poor, largely rural, country with a population of more than 150 million. Although health services remain limited, much has been achieved among the young, but with little care from outside the family for the growing population of the rural elderly. The present study was designed to identify important difficulties in functional capacity in the elderly living in villages under the care of Gonoshasthaya Kendra (GK), a community development organization which provides primary health care through paramedics trained for 2 years within GK. At the time of the survey GK was responsible for the health care of 600 villages with a population of some 1.5 million. Rural health care was administered through 16 sub-centres, administering 40 health centres, from which a paramedic was assigned responsibility for each village, providing front line care.

Our aim was to collect information that would serve to improve the management of disability in the elderly. The WHO Study on global AGEing and adult heath (SAGE) has developed interview schedules to collect data on ageing, which have been used in some less developed countries including one area of Bangladesh [1], but the schedules appeared too complex for our goal of identifying interventions that might be helpful in poor rural communities. We adopted instead the set of 6 disability questions developed by the ‘Washington Group ‘, covering vision, hearing, remembering or concentrating, walking or climbing stairs, self care (washing or dressing) and communicating [2]. We expanded these to help us better understand the circumstances in which difficulties occurred and might be managed. We then used this tool in a survey of elderly villagers living in 535 villages that had been under the care of GK since 2005, when a GK house-to-house census had been conducted by the paramedic assigned to each village.

## Methods

### Target population

1) Conceptual. The survey was designed to include all those still living in 2010 in the village recorded in the 2005 GK census and with a recorded census birth year of 1945 or earlier (i.e. 65 years or greater at the time of the survey).

2) Pragmatic (survey population). All residents of each village believed to be 60 years or older were identified. In the absence of any birth certification true age was difficult to ascertain, either in the census or survey; a standard protocol was used, estimating age from historic events.

3) Matched sub-population. Information from the 2005 census and 2010 survey were matched on household number and sex for all those recorded with a birth before 1945 in the census, thus approximating the initial design.

### Survey card

The survey card (Additional file 1) devised was in 5 parts, demographic (age, sex, marital status, age of living spouse), difficulties with activities of daily life (disability: questions 1-12), health problems (ill-health: Q13), resting tremor, as a coarse screen for Parkinson’s disease (Q14) and help received and needed (Q15 and 16). Questions 1 and 2 on the card and the response scale for all disability questions (‘no problem’ to ‘can’t do it at all’) were taken directly from the Washington Group questions^1^ but the remaining 10 disability questions were elaborated to help identify barriers that might be susceptible to intervention. Respondents were asked to provide their own perception of degree of difficulty but paramedics were asked to record whether this was importantly underrated. The health problems listed were those felt by GK physicians (ZC, RH) to be the most troublesome among elderly villagers: space was left to record ‘other’ problems.

The final English version was translated into Bangla and back-translated before being piloted for comprehension in villages that had come under the care of GK since 2005.

### Administration

Starting in November 2009, the paramedic in each village conducted a house-to-house survey to compile a list of villagers believed to be aged 60 or greater. She then sought to interview all listed, recording the reason for any failure to do so. Supervisors re-interviewed about 10% of respondents to ensure that the interview had indeed been conducted. Cards were checked for completeness locally and returned to the GK research unit for coding and data entry. Responses to the disability and ill-health questions (Q1-14) were entered as recorded. Up to three responses were coded for open-ended items. Data collection was completed in May 2010.

### Matching to census

Survey respondents were matched to the census data , collected by paramedics in a house-to-house survey in 2005, on village, household number and sex Where a match was achieved, census year of birth, educational level, socioeconomic status (used by GK to determine payment), occupation in 2005 and smoking habit (yes/no) were added to the survey data file.

### Statistical methods

Three composite scores were calculated, the total number of disabilities coded as either 3 or 4 (range 0-12), a total disability score (the sum of codes 1-4 on all 12 items: range 12- 48) and the total number of boxes checked (from joints to ‘other’) at Q13 (range 0-10).

The demographics of the 2 populations (survey and census-matched) were compared and the frequency of reporting each disability and health problem examined by age and sex. Disability was considered to be present only for those reporting that they had either ‘much difficulty’ (code 3) or ‘could not do it at all’ (code 4) on Q1-12. The relation of each disability to age, family structure (living spouse) and census information on poverty, literacy, employment and smoking was examined by logistic regression, stratified by sex. The model also included the health problems listed at Q13 (except prolapse, applicable to women only) and at Q14. In these regressions the effect of each factor (present versus absent) was calculated in a model containing all potential predictors and confounders. The relation of total disability score (range 12-48, log-transformed to reduce skew) to health problems as examined by linear regression. Help received and needed was examined by age, sex and extent of disability.

There were very few missing values on items other than age. In analyses including age missing values were excluded: those reporting an age <60 years (but believed to be ≥60 years) were included as a distinct group. The analysis was carried out using SPSS/PASW Statistics 18.

## Results

### Participation and matching

Survey cards were completed and entered for 43417 residents. Non-completion was recorded for 12969, with 54 recorded as refused, 5821 as died before an interview could be completed, 2006 as having moved out of the village and 1496 as still living there but never found at home. No reason was given for non-completion for 3592. In the absence of an independent nominal roll there was some uncertainty about the true size of the target population, but the response rate estimated from these figures (excluding those who had died or moved away) was 89.4% (43417/48559).

Of the 43417 interviewed, 17346 (40%) were successfully matched to a villager of the same sex at the same address in the census data of 2005, with a census birth year ≤ 1945: scrutiny of the names recorded at the census and survey in a random sample of matched records showed a very high concordance (>95%). Because of changes in household numbering no matching was possible for 70 villages. Among those in the remaining 465 villages, subjects not matched either did not appear in the census data for that village (as would happen if they had moved into the village since 2005 or had been omitted in error from the census) or appeared in the census but with a different household number or with a birth year > 1945.

### The study populations

The age and sex reported in the survey are shown in Table 1 for all responders and for the subgroup matched to the census, together with the number of disabilities and health problems reported. The expected deficit (implied by matching on birth ≤ 1945) was evident in the age group 60 < 65 years in the census-matched subpopulation. Women were younger than men (were more often <75 years) and more likely to be widowed. The proportion with no disability or health problem was very similar in the two populations. Overall nearly three quarters of the respondents reported that they had no serious difficulty with any of the functions listed, but the proportion with difficulties increased steadily with age. In those aged 85 years of greater 55% (1796/3620) had ‘much difficulty’ with at least one functional capacity. In this elderly population as a whole <3% reported such difficulty on 6 or more capacities, but this rose to 14.6% (477/3620) in those ≥85 years. Health problems were reported more frequently than ‘much difficulty’ with functional capacities. Almost all respondents (92.7%) reported at least one health problem that made life difficult.

**Table 1:** Distributions of age, sex, marital status, disability and health problems in the survey population and census-matched subpopulation

	**All survey respondents (N = 43417)**				**Respondents matched to census (N = 17346)**			
	**Men**		**Women**		**Men**		**Women**	
	**N**	**%**	**N**	**%**	**N**	**%**	**N**	**%**
**Age from survey**								
<60	1369	6.2	1653	7.7	593	6.2	670	8.5
60 < 65	3836	17.5	5102	23.7	1036	10.9	1246	15.8
65 < 75	5043	23.0	5628	26.2	2261	23.7	2264	28.7
70 < 75	5033	23.0	4215	19.6	2307	24.2	1707	21.6
75 < 80	2705	12.3	1672	7.8	1420	14.9	743	9.4
80 < 85	1988	9.1	1608	7.5	971	10.2	650	8.2
85 < 90	783	3.6	524	2.4	411	4.3	229	2.9
>90	1024	4.7	929	4.3	483	5.1	331	4.2
Unknown	141	0.6	164	0.8	55	0.6	59	0.7
TOTAL	21922	100.0	21495	100.0	9537	100.0	7899	100.0
**Living spouse**								
No	2776	12.7	12199	56.8	1119	11.7	4579	58.0
Yes	19141	87.3	9289	43.2	8416	88.2	3316	42.0
Unknown	5	0.0	7	0.0	2	0.0	4	0.1
TOTAL	21922	100.0	21495	100.0	9537	100.0	7899	100.0
**Number of disabilities (Q1-12) (much difficulty or worse)**								
None	16717	76.3	15489	72.1	7317	76.7	5704	72.2
One	2815	12.8	3084	14.3	1161	12.2	1054	13.3
Two	1034	4.7	1246	5.8	458	4.8	467	5.9
3-5	779	3.6	973	4.5	348	3.6	390	4.9
6 or more	577	2.6	703	3.2	253	2.7	284	3.6
TOTAL	21922	100.0	21495	100.0	9537	100.0	7899	100.0
**Number of health problems (Q13)**								
None	1959	8.9	1237	5.8	944	9.9	528	6.7
One	4088	18.6	3466	16.1	1995	20.9	1485	18.8
Two	6018	27.5	5781	26.9	2597	27.2	2194	27.8
Three	4537	20.7	4985	23.2	1814	19.0	1684	21.3
4 or more	5320	24.3	6026	28.0	2187	22.9	2008	25.4
TOTAL	21922	100.0	21495	100.0	9537	100.0	7899	100.0

At the census (Table 2) some 90% of these women had been recorded as illiterate as had two thirds of men, with younger respondents being less likely to be illiterate than the very oldest. Very few women were employed at the census, with the most common job coded as a day labourer in all age groups. In men the proportion not working increased with age at census, with about a third of the oldest group of men (>75 years in 2005) being classified as ‘dependent’. Farming was by far the most common occupation among men of all ages. Classification by socioeconomic group depended on the assets of the household, with few coded as ‘very poor or destitute’. About half the men but few women were smokers at the time of the census.

**Table 2:** Distributions of education, occupation, socioeconomic group and smoking by age (from census year of birth) and sex: census-matched subpopulation (N = 17436)

	**Men**										**Women**									
	**Age (years)**										**Age (years)**									
	**65-69**		**70-74**		**75-79**		**> 80**		**Total**		**65-69**		**70-74**		**75-79**		**> 80**		**Total**	
	**N**	**%**	**N**	**%**	**N**	**%**	**N**	**%**	**N**	**%**	**N**	**%**	**N**	**%**	**N**	**%**	**N**	**%**	**N**	**%**
**Education**																				
Illiterate	2287	63.4	1628	66.0	1349	70.2	1146	74.3	6410	67.2	2890	87.4	1874	90.1	1337	93.6	1029	95.0	7130	90.3
<5 years	186	5.2	117	4.7	88	4.6	66	4.3	457	4.8	126	3.8	53	2.5	30	2.1	9	0.8	218	2.8
5 years	533	14.8	340	13.8	218	11.3	170	11.0	1261	13.2	182	5.5	88	4.2	36	2.5	31	2.9	337	4.3
>5 years	602	16.7	380	15.4	266	13.8	161	10.4	1409	14.8	110	3.3	64	3.1	26	1.8	14	1.3	214	2.7
TOTAL	3608	100.0	2465	100.0	1921	100.0	1543	100.0	9537	100.0	3308	100.0	2079	100.0	1429	100.0	1083	100.0	7899	100.0
**Occupation at census**																				
Dependent/housewife/UE	366	10.1	324	13.1	459	23.9	532	34.5	1681	17.6	3221	97.4	2032	97.7	1399	97.9	1060	97.9	7712	97.6
Farmer	1971	54.6	1394	56.6	966	50.3	710	46.0	5041	52.9	15	0.5	8	0.4	3	0.2	3	0.3	29	0.4
Business	535	14.8	343	13.9	230	12.0	134	8.7	1242	13.0	12	0.4	9	0.4	8	0.6	4	0.4	33	0.4
Day labourer	280	7.8	171	6.9	103	5.4	76	4.9	630	6.6	35	1.1	17	0.8	10	0.7	6	0.6	68	0.9
Service	209	5.8	104	4.2	72	3.7	45	2.9	430	4.5	13	0.4	2	0.1	3	0.2	4	0.4	22	0.3
Craftsman	66	1.8	36	1.5	22	1.1	13	0.8	137	1.4	0	0.0	2	0.1	0	0.0	0	0.0	2	0.0
Other	181	5.0	93	3.8	69	3.6	33	2.1	376	3.9	12	0.4	9	0.4	6	0.4	6	0.6	33	0.4
TOTAL	3608	100.0	2465	100.0	1921	100.0	1543	100.0	9537	100.0	3308	100.0	2079	100.0	1429	100.0	1083	100.0	7899	100.0
**Socioeconomics group (of household)**																				
Destitute (Aw)	44	1.2	32	1.3	20	1.0	23	1.5	119	1.2	78	2.4	66	3.2	42	2.9	29	2.7	215	2.7
Very poor (Ah)	6	0.2	2	0.1	7	0.4	3	0.2	18	0.2	30	0.9	16	0.8	20	1.4	20	1.8	86	1.1
Poor (Ka)	2201	61.0	1439	58.4	1112	59.9	828	53.7	5580	58.5	2035	61.5	1237	59.5	866	60.6	625	57.3	4763	60.3
Middle class (Kha)	1119	31.0	811	32.9	629	32.7	538	34.9	3097	32.5	983	29.7	613	29.5	409	28.6	323	29.8	2328	29.5
Wealthier (Ga)	238	6.6	181	7.3	153	8.0	151	9.8	723	7.6	182	5.5	147	7.1	92	6.4	86	7.9	507	6.4
TOTAL	3608	100.0	2465	100.0	1921	100.0	1543	100.0	9537	100.0	3308	100.0	2079	100.0	1429	100.0	1083	100.0	7899	100.0
**Smoker**																				
No	1501	41.6	1122	45.5	961	50.0	797	51.7	4381	45.9	3244	98.1	2035	97.9	1412	98.8	1068	98.6	7759	98.2
Yes	2107	58.4	1343	54.5	960	50.0	746	48.3	5156	54.1	64	1.9	44	2.1	17	1.2	15	1.4	140	1.8
TOTAL	3608	100.0	2465	100.0	1921	100.0	1543	100.0	9537	100.0	3308	100.0	2079	100.0	1429	100.0	1083	100.0	7899	100.0

### Disability

Reporting of difficulty (code 3 or 4) increased steadily with age for all disabilities, with women more likely than men to report disability at almost every age (Table 3). The ranking of disabilities was very similar at each age, with difficulties in lifting and carrying, seeing, and going for some distance outside the home being rated as ‘much difficulty’ or ‘can’t do at all’ by some 20% to 50% of those aged 85 or greater. The final column in Table 3 shows the number for whom the paramedic reported the difficulty underestimated. These were uniformly low.

**Table 3:** Number (n) reporting ‘much difficulty ‘or ‘can’t do it at all’ for each functional capacity (Q 1-12) by age and sex (N = 43112)

		**<60**		**60 < 65**		**65 < 70**		**70 < 75**		**75 < 80**		**80 < 85**		**> 85**		**TOTAL**		**% under-rated**
		**n**	**%**	**n**	**%**	**n**	**%**	**n**	**%**	**n**	**%**	**n**	**%**	**n**	**%**	**n**	**%**	
Q1 seeing	men	13	0.9	116	3.0	217	4.3	316	6.3	262	9.7	252	12.7	362	20.0	1538	7.1	1.6
	women	34	2.1	218	4.3	285	5.1	413	9.8	211	12.6	302	18.8	379	26.1	1842	8.6	2.1
Q2 hearing	men	2	0.1	61	1.6	75	1.5	142	2.8	116	4.3	151	7.6	204	11.3	751	3.4	0.8
	women	10	0.6	96	1.9	135	2.4	213	5.1	91	5.4	139	8.6	230	15.8	914	4.3	1.1
Q3 getting up	men	8	0.6	47	1.2	67	1.3	93	1.8	81	3.0	99	5.0	206	11.4	601	2.8	0.2
	women	7	0.4	60	1.2	74	1.3	130	3.1	72	4.3	140	8.7	228	15.7	711	3.3	0.3
Q4 standing	men	9	0.7	36	0.9	75	1.5	101	2.0	94	3.5	118	5.9	223	12.3	656	3.0	0.3
	women	6	0.4	43	0.8	68	1.2	137	3.3	79	4.7	161	10.0	283	19.5	777	3.6	0.2
Q5 walking	men	4	0.3	34	0.9	63	1.2	87	1.7	75	2.8	93	4.7	189	10.4	545	2.5	0.2
	women	7	0.4	42	0.8	57	1.0	119	2.8	68	4.1	141	8.8	227	15.6	661	3.1	0.2
Q6 go outside	men	9	0.7	74	1.9	142	2.8	201	4.0	169	6.2	202	10.2	354	19.6	115	5.3	0.3
	women	12	0.7	101	2.0	191	3.4	303	7.2	174	10.4	293	18.2	426	29.3	1500	7.0	0.3
Q7 washing	men	2	0.1	37	1.0	70	1.4	92	1.8	74	2.7	114	5.7	213	11.8	602	2.8	0.1
	women	10	0.6	38	0.7	72	1.3	127	3.0	79	4.7	152	9.4	253	17.4	731	3.4	0.2
Q8 lavatory	men	4	0.3	35	0.9	70	1.4	84	1.7	68	2.5	92	4.6	196	10.8	549	2.5	0.1
	women	6	0.4	41	0.8	63	1.1	122	2.9	77	4.6	139	8.6	228	15.7	676	3.2	0.1
Q9 understanding	men	11	0.8	65	1.7	123	2.4	153	3.0	127	4.7	161	8.1	259	14.3	899	4.1	0.4
	women	10	0.6	117	2.3	201	3.6	230	5.5	124	7.4	169	10.5	283	19.5	1134	5.3	0.5
Q10 remembering	men	4	0.3	43	1.1	92	1.8	110	2.2	95	3.5	112	5.6	179	9.9	635	2.9	0.6
	women	12	0.7	78	1.5	119	2.1	158	3.7	84	5.0	129	8.0	228	15.7	808	3.8	0.6
Q11 lifting/carrying	men	51	3.7	487	12.7	699	13.9	792	15.7	609	22.5	540	27.2	672	37.2	3850	17.7	1.5
	women	105	6.4	700	13.7	857	15.2	991	23.5	483	28.9	592	36.8	702	48.7	4430	20.8	1.7
Q12 getting food	men	22	1.6	81	2.1	83	1.6	108	2.1	75	2.8	73	3.7	87	4.8	529	2.4	1.3
	women	36	2.2	101	2.0	115	2.0	122	2.9	66	3.9	84	5.2	118	8.1	642	3.0	1.6
TOTAL N	men	1369		3836		5043		5033		2705		1988		1807		21781		-
	women	1653		5102		5628		4215		1672		1608		1453		21331		-

The reporting of troublesome heath conditions followed a similar pattern to that for disability (Table 4) with only uterine prolapse and sexual difficulties not showing increase with age, in women. Painful joints were by far the most common symptom, with little increase in the proportion reporting this symptom once the age of 70 years had been reached. In those ≥85 years chest pain and urinary incontinence were, for both men and women, the second and third most common condition. The recoding of ‘other’ problems was not related to age. Responses to this question had been coded to capture reports of diabetes, hypertension, digestive system problems and back pain, but the number reporting each condition was small.

**Table 4:** Number (n) reporting each health problem (Q 13-14) by age (years) and sex (N = 43,112)

		**<60**		**60 < 65**		**65 < 70**		**70 < 75**		**75 < 80**		**80 < 85**		**>85**		**TOTAL**	
		**n**	**%**	**n**	**%**	**n**	**%**	**n**	**%**	**n**	**%**	**n**	**%**	**n**	**%**	**n**	**%**
Pain in joints	men	763	55.7	2710	70.6	3704	73.4	3811	75.7	2001	74.0	1528	76.9	1366	75.6	15883	72.9
	women	1136	68.7	3995	78.3	4573	81.3	3539	84.0	1380	82.5	1384	86.1	1211	83.4	17218	80.7
Chest pain	men	386	28.2	1466	38.2	2041	40.5	2155	42.8	1109	41.0	868	43.7	751	41.6	8776	40.3
	women	597	36.1	2298	45.0	2547	45.3	2007	47.6	789	47.2	824	51.2	705	48.6	9767	45.8
Breathing problems	men	122	8.9	591	15.4	883	17.5	1115	22.2	648	24.0	544	27.4	534	29.6	4437	20.4
	women	156	9.4	714	14.0	794	14.1	712	16.9	306	18.3	344	21.4	352	24.2	3378	15.8
Urinary incontinence	men	143	10.4	706	18.4	1117	22.1	1425	28.3	827	30.6	683	34.4	726	40.2	5627	25.8
	women	293	17.7	1309	25.7	1553	27.6	1426	33.8	597	35.7	653	40.6	637	43.9	6486	30.3
Uterine prolapse	men	-	-	-	-	-	-	-	-	-	-	-	-	-	-	-	-
	women	99	6.0	247	4.8	352	6.3	255	6.0	120	7.2	109	6.8	101	7.0	1283	6.0
Depression	men	87	6.4	630	16.4	1028	20.5	1053	20.9	592	21.9	491	24.7	478	26.5	4359	20.0
	women	205	12.4	1209	23.7	1335	23.7	1084	25.7	426	25.5	475	29.5	403	27.8	5137	24.1
Stroke/paralysis	men	10	0.7	125	3.3	177	3.5	194	3.9	132	4.9	111	5.6	134	7.4	883	4.1
	women	19	1.1	162	3.2	183	3.3	186	4.1	93	5.6	102	6.3	117	8.1	862	4.0
Itching	men	183	13.4	696	18.1	1059	21.0	1080	21.5	680	25.1	486	24.4	493	27.3	4677	21.5
	women	310	18.8	1067	20.9	1238	22.0	951	22.6	366	21.9	398	24.8	361	24.9	4691	22.0
Sexual difficulties	men	84	6.1	174	4.5	308	6.1	394	7.8	262	9.7	202	10.2	264	14.6	1688	7.8
	women	98	5.9	224	4.4	238	4.7	136	3.2	49	2.9	41	2.5	26	1.8	812	3.8
Other problems	men	531	38.8	1393	36.3	1840	36.5	1772	35.2	950	35.1	688	34.6	656	36.3	7830	36.0
	women	688	41.6	2069	40.6	2188	38.9	1562	37.1	598	35.8	585	36.4	566	39.0	8258	38.7
Hands shaking at rest	men	18	1.3	120	3.1	209	4.1	288	5.7	210	7.8	198	10.0	262	14.5	1305	6.0
	women	36	2.2	193	3.8	253	4.5	300	7.1	133	8.0	191	11.9	223	15.4	1329	6.2
TOTAL N	men	1369		3836		5043		5033		2705		1988		1806		21780	
	women	1653		5102		5628		4215		1672		1608		1452		21330	

Results of 12 logistic regression analyses relating specific disability to socio-demographic and heath conditions showed each disability remained significantly related to increasing age in the full model (except for ‘getting enough to eat ‘for men) (Tables 5 and 6). Widowhood (no living spouse) was associated with the reporting of all but one disability for women and for 9 /12 disabilities for men. In contrast, the small number of ‘very poor or destitute’ were not at greater risk (compared to the wealthiest in these villages), except for not getting enough to eat. Illiteracy was most strongly related, for both men and women, with difficulties seeing and hearing, going outside and lifting and carrying heavy loads. A man not working at the census was more likely to be disabled at the time of the survey (having adjusted for age), being more at risk on 7 of the 12 functional capacities. Smoking was not related to disability.

**Table 5:** Relation of disabilities to social factors and illness (Functional capacities 1-6): multivariate logistic regression (N = 17436)

	**Sex**	**Disability**											
		**Q1 seeing**		**Q2 hearing**		**Q3 getting up**		**Q4 standing**		**Q5 walking**		**Q6 go outside**	
		**OR**	**95% CI**	**OR**	**95% CI**	**OR**	**95% CI**	**OR**	**95% CI**	**OR**	**95% CI**	**OR**	**95% CI**
**Sociodemographic**													
Age from census (continuous variable)	men	1.04	1.03-1.05	1.04	1.02-1.05	1.06	1.04-1.07	1.06	1.05-1.08	1.05	1.03-1.07	1.05	1.04-1.63
	women	1.03	1.02-1.04	1.04	1.03-1.73	1.04	1.03-1.06	1.05	1.03-1.06	1.04	1.02-1.06	1.04	1.03-1.06
No living spouse	men	1.37	1.14-1.64	1.28	1.02-1.61	1.21	0.91-1.60	1.21	0.93-1.57	1.21	0.90-1.62	1.51	1.24-1.85
	women	1.42	1.23-1.65	1.45	1.21-1.73	1.51	1.24-1.85	1.61	1.32-1.98	1.48	1.21-1.82	1.68	1.41-2.00
Very poor/destitute*	men	1.20	0.65-2.22	1.84	0.82-4.11	0.37	0.09-1.54	0.64	0.21-1.95	0.39	0.09-1.65	0.42	0.16-1.93
	women	1.34	0.83-2.18	1.00	0.50-2.00	1.29	0.58-2.85	1.17	0.54-2.52	1.02	0.43-2.42	0.81	0.46-1.43
Illiterate	men	1.34	1.11-1.62	1.31	1.01-1.72	1.10	0.80-1.49	1.31	0.85-1.53	1.18	0.85-1.63	1.23	0.99-1.54
	women	1.70	1.20-2.40	1.42	0.89-2.29	0.97	0.60-1.57	1.45	0.86-2.48	1.27	0.74-2.16	1.75	1.18-2.61
No job at census	men	1.33	1.10-1.61	1.62	1.24-2.10	1.29	0.94-1.77	1.31	0.97-1.77	1.46	1.06-2.01	1.35	1.08-1.70
	women	1.29	0.74-2.22	0.83	0.42-1.64	1.38	0.52-3.32	1.45	0.57-3.65	1.13	0.45-2.55	1.61	0.80-3.22
Smoker at census	men	0.88	0.75-1.03	1.00	0.80-1.25	0.84	0.64-1.10	0.95	0.73-1.23	0.86	0.65-1.14	1.03	0.85-1.25
	women	0.50	0.23-1.09	0.49	0.15-1.56	0.61	0.18-2.07	0.57	0.17-1.96	0.67	0.20-2.31	0.70	0.31-1.57
**Disease/condition**													
Pain in joints	men	1.13	0.93-1.37	1.17	0.89-1.55	1.00	0.73-1.37	1.06	0.79-1.44	1.21	0.87-1.70	1.20	0.95-1.51
	women	1.30	1.04-1.63	1.02	0.75-1.38	1.05	0.75-1.47	0.84	0.61-1.15	0.81	0.58-1.13	1.26	0.99-1.61
Chest pain	men	1.25	1.05-1.47	1.46	1.15-1.85	1.00	0.75-1.33	0.86	0.65-1.13	0.88	0.66-1.19	0.97	0.90-1.19
	women	1.28	1.08-1.51	1.31	1.03-1.66	0.64	0.48-0.85	0.68	0.52-0.89	0.73	0.54-0.97	0.85	0.71-1.03
Breathing problem	men	1.48	1.24-1.77	1.31	1.02-1.68	1.50	1.12-2.01	1.73	1.32-2.28	1.46	1.08-1.97	1.45	1.18-1.79
	women	1.30	1.06-1.58	1.15	0.87-1.51	1.32	0.96-1.81	1.29	0.95-1.75	1.26	0.91-1.74	1.23	0.98-1.53
Incontinence	men	1.52	1.28-1.81	1.69	1.32-2.15	1.61	1.21-2.15	1.58	1.20-2.08	1.58	1.17-2.12	1.87	1.52-2.29
	women	1.41	1.19-1.67	1.54	1.21-1.95	1.81	1.37-2.39	2.06	1.58-2.69	1.83	1.38-2.44	1.80	1.49-2.18
Depression	men	1.56	1.30-1.88	1.32	1.02-1.72	1.60	1.18-2.16	1.46	1.09-1.95	1.69	1.24-2.29	1.63	1.31-2.01
	women	1.53	1.28-1.83	1.26	0.98-1.62	1.38	1.03-1.85	1.42	1.08-1.87	1.29	0.96-1.74	1.48	1.21-1.80
Paralysis	men	1.09	0.78-1.53	1.33	0.87-2.04	9.06	6.61-12.41	8.53	6.28-11.58	10.20	7.42-14.02	4.90	3.75-6.41
	women	1.79	1.33-2.41	0.98	0.62-1.54	13.68	10.10-18.53	11.56	8.56-15.60	13.67	10.04-18.61	6.10	4.68-7.95
Itching	men	0.99	0.83-1.19	0.87	0.67-1.14	0.77	0.56-1.06	0.75	0.55-1.01	0.71	0.51-0.99	0.91	0.73-1.13
	women	0.91	0.75-1.10	1.33	1.04-1.70	0.78	0.57-1.07	0.92	0.68-1.24	0.88	0.64-1.22	0.96	0.78-1.19
Other	men	1.57	1.34-1.84	1.37	1.09-1.72	1.38	1.05-1.82	1.44	1.11-1.86	1.46	1.11-1.94	1.42	1.17-1.72
	women	1.62	1.38-1.90	1.52	1.22-1.89	1.53	1.18-1.98	1.59	1.24-2.04	1.50	1.15-1.96	1.51	1.26-1.80
Shaking at rest	men	1.97	1.53-2.53	2.59	1.90-3.55	3.92	2.84-5.41	3.89	2.85-5.30	4.4	3.21-6.14	2.89	2.23-3.74
	women	2.58	2.05-3.25	3.17	2.36-4.25	3.14	2.26-4.35	3.57	2.62-4.86	3.51	2.53-4.88	2.53	1.97-3.25

· Relative to wealthier.

**Table 6:** Relation of disabilities to social factors and illness (Functional capacities 7-12): multivariate logistic regression (N = 17436)

	**Sex**	**Disability**											
		**Q7 bath**		**Q8 lavatory**		**Q9 understand**		**Q10 Memory**		**Q11 lifting**		**Q12 food**	
		**OR**	**95% CI**	**OR**	**95% CI**	**OR**	**95% CI**	**OR**	**95% CI**	**OR**	**95% CI**	**OR**	**95% CI**
**Sociodemographic**													
Age from census	men	1.05	1.03-1.06	1.05	1.03-1.07	1.04	1.03-1.06	1.04	1.03-1.06	1.02	1.02-1.03	1.01	0.99-1.06
	women	1.04	1.03-1.06	1.05	1.03-1.07	1.04	1.03-1.05	1.05	1.03-1.06	1.03	1.02-1.04	1.03	1.01-1.05
No living spouse	men	1.42	1.13-1.80	1.36	1.06-1.74	1.50	1.22-1.84	1.51	1.20-1.89	1.41	1.21-1.63	1.38	1.04-1.84
	women	1.47	1.21-1.80	1.55	1.27-1.91	1.37	0.55-2.04	1.39	1.15-1.68	1.47	1.31-1.66	1.29	1.02-1.62
Very poor/destitute	men	0.55	0.16-1.93	0.39	0.10-1.59	0.89	0.37-2.14	0.56	0.20-1.61	0.55	0.32-0.89	3.86	1.47-10.16
	women	1.45	0.67-3.13	1.12	0.50-2.49	1.06	0.55-2.04	0.82	0.42-1.62	0.71	0.50-1.03	5.14	2.33-11.35
Illiterate	men	1.08	0.80-1.47	1.00	0.73-1.36	1.04	0.82-1.31	1.21	0.91-1.62	1.35	1.19-1.54	1.03	0.71-1.49
	women	1.19	0.73-1.95	1.35	0.78-2.33	1.96	1.24-3.10	1.91	1.11-3.30	1.53	1.22-1.91	1.39	0.74-2.63
No job at census	men	1.53	1.12-2.07	1.53	1.11-2.11	1.48	1.16-1.89	1.07	0.79-1.45	1.14	0.98-1.32	1.10	0.73-1.64
	women	1.00	0.45-2.22	1.17	0.47-2.93	0.78	0.43-1.43	0.62	0.33-1.19	1.18	0.79-1.77	0.51	0.28-0.93
Smoker at census	men	1.08	0.83-1.41	1.06	0.80-1.41	1.07	0.87-1.32	0.83	0.64-1.06	1.11	0.99-1.24	0.91	0.66-1.26
	women	1.57	0.69-3.58	0.71	0.21-2.39	0.79	0.34-1.82	0.78	0.29-2.18	0.60	0.35-1.00	0.91	0.32-2.29
**Disease/condition**													
Pain in joints	men	1.05	0.77-1.44	1.21	0.80-1.41	1.15	0.90-1.49	1.08	0.80-1.45	1.15	1.01-1.32	1.25	0.82-1.90
	women	0.90	0.65-1.23	1.05	0.74-1.49	1.15	0.88-1.51	0.88	0.64-1.20	1.31	1.12-1.53	0.90	0.61-1.33
Chest pain	men	0.79	0.60-1.05	0.82	0.61-1.11	1.26	1.02-1.56	0.98	0.75-1.27	1.11	0.98-1.23	1.26	0.90-1.76
	women	0.72	0.55-0.93	0.68	0.51-0.91	1.10	0.89-1.35	0.99	0.77-1.28	1.06	0.94-1.70	1.17	0.86-1.60
Breathing problem	men	1.53	1.16-2.03	1.36	1.01-1.84	1.06	0.84-1.34	1.22	0.93-1.62	1.30	1.14-1.49	1.92	1.37-2.67
	women	1.24	0.91-1.69	0.99	0.70-1.39	1.25	0.98-1.60	0.82	0.60-1.13	1.15	0.98-1.34	1.66	1.19-2.31
Incontinence	men	1.66	1.26-2.20	1.75	1.31-2.35	1.55	1.24-1.93	1.55	1.19-2.03	1.71	1.51-1.94	1.99	1.42-2.78
	women	1.63	1.25-2.14	1.66	1.25-2.21	1.30	1.05-1.61	1.75	1.36-2.24	1.37	1.21-1.56	1.11	0.81-1.52
Depression	men	1.72	1.29-2.29	1.70	1.26-2.31	1.59	1.26-2.01	1.83	1.39-2.42	1.61	1.40-1.84	1.87	1.32-2.64
	women	1.37	1.03-1.82	1.44	1.07-1.93	1.17	0.93-1.47	1.58	1.22-2.05	1.39	1.21-1.59	2.19	1.60-3.00
Paralysis	men	7.97	5.83-10.89	8.42	6.11-11.62	1.74	1.22-2.49	2.56	1.76-3.72	2.47	1.97-3.09	2.06	1.26-3.36
	women	11.64	8.61-15.72	12.23	8.94-16.74	2.12	1.51-2.97	3.18	2.23-4.52	3.30	2.60-4.19	3.08	2.01-4.72
Itching	men	0.84	0.62-1.14	0.75	0.55-1.04	1.38	1.11-1.73	1.00	0.76-1.33	1.04	0.91-1.19	1.14	0.81-1.61
	women	0.85	0.63-1.15	0.93	0.67-1.27	1.41	1.51-2.97	1.13	0.93-1.54	1.10	0.96-1.27	1.45	1.06-1.99
Other	men	1.57	1.21-2.04	1.48	1.12-1.95	1.12	0.91-1.38	1.22	0.95-1.57	1.62	1.45-1.82	1.65	1.20-2.26
	women	1.50	1.16-1.92	1.44	1.10-1.88	1.29	1.06-1.57	1.21	0.95-1.53	1.82	1.62-2.04	1.48	1.11-1.98
Shaking at rest	men	5.41	4.00-7.30	4.64	3.37-6.40	2.38	1.78-3.17	3.97	2.93-5.39	2.49	2.05-3.01	2.21	1.44-3.40
	women	3.43	2.51-4.69	3.27	2.35-4.53	2.41	1.83-3.18	3.63	2.70-4.87	2.50	2.06-3.06	1.91	1.28-2.86

The relation between disability and troublesome health conditions varied markedly with the type of ill-health. Painful joints, although the most common complaint, were related only to difficulty lifting whereas hemiplegia and resting tremor were associated with increased risk of reporting every dimension of disability. In men, but not in women, breathing problems were commonly associated with disability. Chest pain showed little consistent relation to disability. Further analysis indicated a strong relation between depression and reports of chest pain: 55.9% (5345/9556) of those saying that depression often made life difficult reported chest pain compared to 39.3% (13307/33861) of those not reporting depression. Inclusion of depression in the model attenuated the bivariate relation between chest pain and, for example, difficulty walking in the home. Overall, the relation between depression and reported disabilities was striking (Tables 5 and 6): a subject who was ‘very often depressed’ was more likely to report difficulties on each of the disability questions (except hearing for women). The relation of disability to urinary incontinence was at least as strong, with both men and women who reported troublesome urinary incontinence being more likely to report each disability (except, for women, not getting enough food). In all age groups there was a close relation between urinary incontinence and depression, with depression reported overall in 41.0% of those who reported troublesome urinary incontinence but only 14.6% of those who did not. In a linear regression in which total disability score was the dependent variable, urinary incontinence was more strongly related to overall disability than any health problem except hemiplegia tremor and resting tremor (Table 7).

**Table 7:** Relation of total disability (log score) to health problems (N = 43112)

	**Standardised βeta**	**t**	**p<**
Pain in joints	0.040	9.47	0.000
Chest pain	0.058	13.54	0.000
Breathing problems	0.050	12.02	0.000
Incontinence	0.119	27.53	0.000
Depression	0.101	23.54	0.000
Paralysis	0.161	38.90	0.000
Itching	0.030	7.20	0.000
Other	0.089	21.93	0.000
Shaking at rest	0.132	31.80	0.000
Sex: female	0.062	13.20	0.000
No living spouse	0.054	11.23	0.000
Age (continuous)	0.318	73.23	0.000

### Help received and needed

More than half the oldest group (over 85 years) had some help from a family member (Table 8): help from someone outside the family was mentioned by only 39: of 11211 villagers with ‘much difficult’ on at least one functional capacity, only 15 reported getting help outside the family. For women help was most commonly from a daughter-in-law, with mobility and bathing the most common assistance. In a logistic regression analysis, with any help reported (or not) as the outcome, help received was reported somewhat more frequently by women, by those with no living spouse, those who were older and those with a higher disability score (Table 9).

**Table 8:** Types of help obtained and needed (Q15 and Q16) by age (years) and sex

		**< 60**		**60 < 65**		**65 < 70**		**70 < 75**		**75 < 80**		**80 < 85**		**> 85**		**Total**	
		**n**	**%**	**n**	**%**	**n**	**%**	**n**	**%**	**n**	**%**	**n**	**%**	**n**	**%**	**n**	**%**
**Help Given**																	
Some help given	men	96	7.0	824	21.5	1113	22.1	1435	28.5	837	30.9	820	41.2	949	52.3	6074	27.9
	women	216	13.1	1291	25.3	1351	24.0	1415	33.6	613	36.7	779	48.4	822	56.6	6487	30.4
By spouse	men	65	4.7	466	12.1	609	12.1	710	14.1	43.9	16.2	343	17.2	324	17.9	2955	13.6
	women	40	2.4	85	1.7	74	1.3	59	1.4	22	1.3	18	1.1	17	1.2	315	1.5
By daughter-in-law	men	18	1.3	116	4.3	263	5.2	455	9.0	294	10.9	338	17.0	510	28.2	2044	9.1
	women	90	5.4	721	14.1	838	14.9	877	20.8	387	23.1	496	30.8	548	37.7	3957	18.6
By son	men	17	1.2	186	4.8	253	5.0	282	5.6	127	4.7	145	7.3	146	8.1	1156	5.3
	women	68	4.1	345	6.8	279	5.0	278	6.6	115	6.9	115	7.2	125	8.6	1325	6.2
By daughter	men	16	1.2	82	2.1	123	2.4	104	2.1	71	2.6	56	2.8	69	3.8	521	2.4
	women	46	2.8	198	3.9	191	3.4	190	4.5	85	5.1	112	7.0	118	8.1	940	4.4
By grandchild	men	0	0.0	15	0.4	14	0.3	53	1.1	25	0.9	46	2.3	66	3.7	219	1.0
	women	6	0.4	58	1.1	91	1.6	124	2.9	65	3.9	110	6.8	124	8.5	578	2.7
**Types of help**																	
Mobility	men	16	1.2	148	3.9	246	4.9	307	6.1	222	8.2	265	13.3	363	20.1	1567	7.2
	women	39	2.4	225	4.4	330	5.9	401	9.5	203	12.1	299	18.6	371	25.5	1868	8.8
Bathing	men	19	1.4	121	3.2	204	4.0	308	6.1	241	8.9	269	13.5	424	23.5	1586	7.3
	women	21	1.3	163	3.2	234	4.2	373	8.8	203	12.1	294	18.3	422	29.0	1710	8.0
Feeding	men	26	1.9	160	4.2	244	4.8	317	6.3	196	7.2	198	10.0	271	15.0	1412	6.5
	women	41	2.5	224	4.4	258	4.6	321	7.6	143	8.6	191	11.9	258	17.8	1436	6.7
Washing clothes	men	14	1.0	73	1.9	121	2.4	196	3.9	147	5.4	139	7.0	228	12.6	918	4.2
	women	16	1.0	100	2.0	136	2.4	213	5.1	108	6.5	179	11.1	209	14.4	961	4.5
**Some help needed**	men	620	45.3	2382	62.1	3202	63.5	3309	65.7	1832	66.7	1397	70.3	1273	70.4	14015	64.3
	women	859	52.0	3504	68.7	3871	68.8	3002	71.2	1192	71.3	1189	73.9	1074	73.9	14691	68.9
**Types of help**																	
Treatment	men	373	27.2	1578	41.1	2090	41.4	2056	40.9	1231	45.5	838	42.2	834	46.2	9000	41.3
	women	565	34.2	2322	45.5	2424	43.1	1833	43.5	753	45.0	732	45.5	703	48.4	9332	43.7
Financial	men	214	15.6	751	19.6	1020	20.2	1081	21.5	518	19.1	459	23.1	364	20.1	4404	20.2
	women	241	14.6	1071	21.0	1329	23.6	1052	25.0	394	23.6	402	25.0	332	22.8	4821	22.6
Prosthesis	men	36	2.6	101	2.6	128	2.5	163	3.2	84	3.1	70	3.5	62	3.4	644	3.0
	women	52	3.1	111	2.2	143	2.5	131	3.1	45	2.7	58	3.6	59	4.1	599	2.8
**Total N**	men	1369		3836		5043		5033		2705		1988		1807		21781	
	women	1653		5102		5628		4215		1672		1608		1453		21331	

**Table 9:** Any help received by need (logistic regression N = 43112)

	**Odds Ratio**	**95% CI**
Indication of Need		
No living spouse	1.13	1.07-1.19
Female	1.10	1.04-1.15
Age (continuous)	1.04	1.03-1.04
Disabilities (much difficulty)		
None	1	-
One	1.71	1.61-1.81
Two	2.83	2.59-3.09
3-5	4.47	4.03-4.96
6 or more	14.94	12.66-17.65

Nearly two thirds of both men and women in the survey reported that they were in need of help. The help specified was most usually treatment for a medical condition or financial aid (Table 8).

## Discussion

This survey of disability in some 43,000 villagers believed to be aged ≥60 years found that only a minority (26%) reported ‘much difficulty’ on any of 12 functional capacities. The proportion increased markedly with age and amongst the most elderly (≥85 years) there were widespread problems, in lifting and carrying, with eyesight and with going outside the house for any distance. It is of note that only 29% of the elderly villagers reported receiving any help from their family members and virtually none had help from outside the family. However those receiving help from the family did appear to be those with the greatest needs.

The study was set up to find ways in which the extent and impact of disabilities could be lessened by appropriate interventions. The high disability rate among those with hemiplegia was expected but the recent introduction by GK of community physiotherapists may help to ensure that a greater proportion of survivors have rapid and appropriate rehabilitation. The comprehensive range of disability among those with a resting tremor is also of interest and would warrant a more focused inquiry: those reporting the symptom here are unlikely to have been formally assessed or treated. Further investigation is also needed of the possible contribution of high levels of manganese (commonly found in drinking water in rural Bangladesh [3]) to Parkinson-like illness [4]. If this were demonstrated, primary prevention of the disease and subsequent disability might be feasible. There is also some scope for intervention to meet the needs of the relatively small group – a total of 1243 – who reported that they would be helped by a prosthesis, mainly to aid mobility or vision. The high rate of disability reported by those with urinary incontinence is of particular interest, not least because of the possibility of intervention to improve its management [5,6]. The direction of causality between the incontinence and the reported disability (and the relation to depression) is likely to be complex. Given that toilet facilities in Bangladeshi village homes are outside the main living quarters, the ability to hold urine may be severely challenged in an elderly person with poor mobility and vision. A program to increase mobility and to improve the management of urinary incontinence would have priority in this population.

The strength of the study lies in the representation of functional difficulties and ill-health in an entire population of elderly rural villagers and in the completeness of the data: there were very few refusals and the paramedics were scrupulous about completing every question. The ability to match a substantial, and apparently representative, sub-group to census data collected 5 years earlier was also a strength of the study, allowing assessment of socio-demographic factors independent of current difficulties. The main weaknesses were the uncertainty about true age and the related difficulty of establishing a definitive list of eligible participants. Also, the data collected, both in the survey and census, failed to catch some elements of importance. While the survey asked about difficulties in understanding speech, for example, it did not ask about difficulties of expression: while the census asked about current smoking habit, it did not include amount smoked, or allow us to identify ex-smokers who had, perhaps, stopped smoking after developing disability, prior to the census. The pattern of causality was also uncertain for other observed relationships such as illiteracy and difficulty carrying heavy loads (where the physical demands may have been greater than for those with education) and the high levels of disability in those men who had already given up work by the time of the census, 5 years previously. Interpretation of the relation between poor functional capacity and reports of very often feeling depressed is also critical to decisions about interventions, designed to reduce both objective incapacity and also feelings of hopelessness. The study did not include objective measures of capacity, but relied on the villager’s own report of degree of difficulty with each dimension: such self-perception of incapacity may be the appropriate metric, although perhaps less so for those with cognitive impairment. It was reassuring that the paramedics very seldom recorded that the degree of disability was under-estimated. The converse – of exaggerating disability – was not explored systematically, but the low proportions reporting ‘much difficulty’, particularly in those below 70 years does not suggest that exaggeration was widespread.

This is not the first study of disability in Bangladesh, although it is by far the largest, covering villages from 4 Divisions of the country. An earlier community based study of some of the same villages found that 50% of those >80 years had physician diagnosed disabilities, most frequently hearing, vision and movement difficulties [7]. Data from Matlab, an area to the south east of Dhaka, was included in the report of the WHO Sage studies, and showed greater disability in women, in older respondents, in people who were single, older, and less educated [1]. The study, which included some 850 subjects ≥70 years, did not report the prevalence of particular disabilities. Other reports from Matlab include an attempt to better understand the value of self-reported health status in older Bangladesh villagers which found, as in the present study, that respondents were more likely to report ill health than disability [8]. The strong relation between incontinence and depression observed here has been widely reported in other populations, including elderly people in Pakistan, with the need for cleanliness in Muslim religious observance being an additional dimension [9-11].

The messages from this study are far reaching. First, at the level of primary health care providers, the results underline the urgent need for programs focusing on the elderly, to alleviate those disabilities that are amenable to intervention and to provide support and care for those with multiple disabilities. Instituting these programs will require development of training programs and health education materials, so that care of the elderly can be successfully integrated into primary health care. Such concentration on the elderly will need new funding, and cannot rely simply on the redistribution of resources away from existing programs, such as those for mothers and children. From the study reported here it is clear that funds are needed to support programs to increase mobility, enhance vision and hearing and to decrease the toll of incontinence and depression found to be so common in these elderly villagers. Alleviating these disabilities will require new approaches to care for the rural elderly, backed by demonstration projects to evaluate the viability and effectiveness of culturally appropriate interventions. Although the study reported here has shown that family support is still provided for many (though not the majority) of these elderly villagers, with rapid urbanization, and the departure of the young and healthy to the cities, family structures for the care of the elderly will surely break down, as has already been shown in China [12,13]. Where young people leave, rural communities will be faced with the need to fill this gap with the provision of community facilities, giving help with feeding and personal care, and aids with vision and mobility to assure accessibility. With such help, the elderly can become more largely self-sufficient, as happens through comprehensive home and social care in wealthy developed counties, in which the maintenance of the elderly at home is seen as a prime goal for social programs. In Bangladesh, the government has begun to recognize the need for social welfare programs for the elderly, but the problems are still substantial, both in Bangladesh and other poor developing countries. Until recently the focus of WHO and donor agencies has been very largely on infants, children and those of reproductive age, but it is no longer defensible to assume either that the rural poor will not survive to old age – they increasingly do – or that younger women in the household will continue to be willing and available to help with basic needs. A new vision is needed in which the residual capacities of the old are nurtured, remediable deficiencies are attacked vigorously and community facilities put in place to reduce the physical, emotional and cognitive isolation of old people living out their years in discomfort and poverty.

## Conclusion

In this study, disabled elderly residents of rural villages in Bangladesh were found to be dependent on the family for help. With family cohesiveness under threat from migration to the city, there is a pressing need for the development and critical evaluation of community-based interventions designed specifically for the elderly in poor rural societies. New approaches to training and practice will be needed to integrate such disability management into primary care.

## Competing interests

The authors declare that they have no competing interests.

## Authors’ contributions

NC, ZC and CMcD conceived the study, developed the design, methodology and measurement tools. RH and MC organized the field work and verification of information accuracy. NC and MC were responsible for data entry and verification. NC and CMcD .planned and executed the data analysis. NC drafted the paper and acts as guarantor. All authors commented critically on the initial draft and have agreed the final text.

## Ethical issues

The proposal for this project was approved by the Health Research Ethics Board of the University of Alberta (Pro00009158 ) and by the Gonoshasthaya Kendra ethics committee. All subjects gave oral informed consent.

## Sources of funding

The work was funded from research funds held at the National Heart and Lung Institute, London.The funding source had no role in the study design, collection, analysis or interpretation, in the writing of the report or in the decision to submit.

## Pre-publication history

The pre-publication history for this paper can be accessed here:

http://www.biomedcentral.com/1471-2458/12/379/prepub

## Supplementary Material

Additional file 1 Survey Card. Click here for file

## References

[B1] Razzaque A Nahar L Akter Khanam M Kim Streatfield P Socio-demographic differentials of adult health indicators in Matlab, Bangladesh: self-rated health, health state, quality of life and disability level Glob Health Action 2010 Supplement 2 70 77 10.3402/gha.v3i0.4618 PMC2958199 20975960

[B2] Madans JH Loeb ME Altman BM Measuring disability and monitoring the UN convention on the rights of persons with disabilities: the work of the Washington Group on disability statistics BMC Public Health 2011 11 (Suppl 4 S4 21624190 10.1186/1471-2458-11-S4-S4 PMC3104217

[B3] Kinniburgh DG, Smedley PL Arsenic contamination of groundwater in Bangladesh BGS Technical Report WC/00/19 2001 British Geological Survey, Keyworth

[B4] Kondakis XG Makris N Leotsinidis M Prinou M Papapetropoulos Health effects of high manganese concentration in drinking water Arch Environ Health 1989 44 175 178 10.1080/00039896.1989.9935883 2751354

[B5] Hay-Smith EJ Dumoulin C Pelvic floor training versus no treatment, or inactive control treatments for urinary incontinence in women Cochrane Database Syst Rev 2006 1 CD005654 16437536 10.1002/14651858.CD005654

[B6] Kim H Yoshida H Suzuki T The effects of multidimensional exercise treatment on community-dwelling elderly Japanese women with stress, urge and mixed urinary incontinence: a randomized controlled trial Int J Nursing Stud 2011 48 1165 1172 10.1016/j.ijnurstu.2011.02.016 21459381

[B7] Hosain GMM Disability problem in a rural area of Bangladesh Bangladesh Med Res Counc Bull 1995 21 24 31 7575340

[B8] Rahman MO Barsky AJ Self-reported health among older Bangladeshis: how good a health indicator is it? Gerontologist 2003 43 856 863 10.1093/geront/43.6.856 14704385

[B9] Yip SK Cardozo L Psychological morbidity and female urinary incontinence Best Pract Res Clin Obstet Gynaecol 2007 21 321 329 10.1016/j.bpobgyn.2006.12.002 17207664

[B10] Ganatra HA Prevalence and predictors of depression among an elderly population of Pakistan Aging Ment Health 2008 12 349 356 10.1080/13607860802121068 18728948

[B11] Wilkinson K Pakistani women's perceptions and experiences of incontinence Nurs Stand 2001 16 33 39 11977796 10.7748/ns2001.10.16.5.33.c3099

[B12] Joseph AE Phillips DR Ageing in rural China: impacts of increasing diversity in family and community resources J Cross Cult Gerontol 1999 14 153 168 10.1023/A:1006658706496 14617890

[B13] Giles J Mu R Elderly parent health and the migration decisions of adult childref: evidence from rural China Demography 2007 44 265 288 10.1353/dem.2007.0010 17583305

