# Supplementary material for: Disability among elderly rural villagers: report of a survey from Gonoshasthaya Kendra, Bangladesh
Source: BMC Public Health. 2012 May 25;12:379. doi: 10.1186/1471-2458-12-379 (PMC3422201; doi:10.1186/1471-2458-12-379)
Supplement: Additional file 1 — Survey Card. [file 1471-2458-12-379-S1.pdf]

Name:………………………………………	**G K Disability Survey, 2009**	Village:…………………………………………
GA: ………… PA: …………..	Male ☐ Female ☐ Age……… Years	Union………… Upazilla:…………………

Husband/wife alive? Yes ☐ No ☐ If yes, age of husband/wife ___years (if >65 mark name on the list)

Do you experience difficulty in doing the following things?	Opinion of senior person				Paramedic: Degree underrated?
	I experience no problem	Yes, I have some difficulty	Yes, I experience much difficulty	I cannot do this at all	√ + ++
1. Any difficulty seeing things? (even with glasses?)					
2. Any difficulty hearing? (Even with Hearing aid)?					
3. Is there Any difficulty in getting up from sitting position without help?					
4. Is there Any difficulty in standing without holding on to something?					
5. Can you walk by yourself within the house?					
6. Can you go outside the house – some distance?					
7. If there is water in a bucket, can you take bath by yourself.					
8. Can you go to the lavatory by yourself?					
9. Do you feel difficulties to listen to or to understand people's speech or what people around are talking about?					
10. Can you remember what you did, eat or who visited you yesterday?					
11. Any difficulty in lifting or carrying heavy things?					
12. Any difficulty in getting enough food?					

13. Do you have any health problem that makes life difficult? Yes ☐ No ☐
- If the answer is yes, which ones are most troublesome?

Pain in different joints ☐	Chest pain ☐	Sexual difficulties ☐
Breathing problem ☐	Very often depressed ☐	Itching ☐
Incontinence (urine) ☐	Prolapse uterus (woman) ☐	One sided paralysis ☐

Any other problem – please describe: ………

14. Do your hands shake even when you are resting? Yes ☐ No ☐ Paramedic-Have you observed this? Yes ☐ No ☐

15. Does someone's help make life easier? Yes ☐ No ☐

If yes, who provides such help (relationship) and how?...

16. Please describe what help you would need to make everyday life more liveable?...

Paramedic comment……

Full name of Paramedic:……………………… Signature of paramedic:………………….Date of Survey (Bangla calendar): …………
Date of Survey (English calendar)…………..
